# A prognostic model for ovarian cancer

**DOI:** 10.1054/bjoc.2001.2030

**Published:** 2001-09

**Authors:** T G Clark, M E Stewart, D G Altman, H Gabra, J F Smyth

**Affiliations:** ICRF Medical Statistics Group, Centre for Statistics in Medicine, Institute of Health Sciences, Old Road, Headington, Oxford, OX3 7LF; ICRF Medical Oncology Unit, Western General Hospital, Edinburgh, EH4 2XU, UK

**Keywords:** ovarian cancer, prognostic model, overall survival

## Abstract

About 6000 women in the United Kingdom develop ovarian cancer each year and about two-thirds of the women will die from the disease. Establishing the prognosis of a woman with ovarian cancer is an important part of her evaluation and treatment. Prognostic models and indices in ovarian cancer should be developed using large databases and, ideally, with complete information on both prognostic indicators and long-term outcome. We developed a prognostic model using Cox regression and multiple imputation from 1189 primary cases of epithelial ovarian cancer (with median follow-up of 4.6 years). We found that the significant (*P*≤ 0.05) prognostic factors for overall survival were age at diagnosis, FIGO stage, grade of tumour, histology (mixed mesodermal, clear cell and endometrioid versus serous papillary), the presence or absence of ascites, albumin, alkaline phosphatase, performance status on the ZUBROD-ECOG-WHO scale, and debulking of the tumour. This model is consistent with other models in the ovarian cancer literature; it has better predictive ability and, after simplification and validation, could be used in clinical practice. http://www.bjcancer.com © 2001 Cancer Research Campaignhttp://www.bjcancer.com


British Journal of Cancer (2001) 85(7), 944–952
© 2001 Cancer Research Campaign
doi: 10.1054/ bjoc.2001.2030, available online at http://www.idealibrary.com on IDEAL

http://www.bjcancer.com

### A prognostic model for ovarian cancer

**TG Clark[1], ME Stewart[2], DG Altman[1], H Gabra[2] and JF Smyth[2]**

[1]ICRF Medical Statistics Group, Centre for Statistics in Medicine, Institute of Health Sciences, Old Road, Headington, Oxford OX3 7LF; [2]ICRF Medical Oncology Unit, Western General Hospital, Edinburgh EH4 2XU, UK

**Summary** About 6000 women in the United Kingdom develop ovarian cancer each year and about two-thirds of the women will die from the disease. Establishing the prognosis of a woman with ovarian cancer is an important part of her evaluation and treatment. Prognostic models and indices in ovarian cancer should be developed using large databases and, ideally, with complete information on both prognostic indicators and long-term outcome. We developed a prognostic model using Cox regression and multiple imputation from 1189 primary cases of epithelial ovarian cancer (with median follow-up of 4.6 years). We found that the significant ($P \leq 0.05$) prognostic factors for overall survival were age at diagnosis, FIGO stage, grade of tumour, histology (mixed mesodermal, clear cell and endometrioid versus serous papillary), the presence or absence of ascites, albumin, alkaline phosphatase, performance status on the ZUBROD-ECOG-WHO scale, and debulking of the tumour. This model is consistent with other models in the ovarian cancer literature; it has better predictive ability and, after simplification and validation, could be used in clinical practice. © 2001 Cancer Research Campaign http://www.bjcancer.com

**Keywords**: ovarian cancer; prognostic model; overall survival

Ovarian carcinoma is the commonest cause of death from gynaecological malignancy in most of the Western world. About 6000 women in the United Kingdom develop ovarian cancer each year and about two-thirds of the women will die from the disease. The age-standardised death rate of 15/100 000 has doubled in the past 70 years, and 5-year survival is just 30% (CRC CancerStats, 2000).

Establishing the prognosis of a woman with ovarian cancer is an important part of her evaluation and treatment. Multiple factors such as age, grade, FIGO stage, residual disease, CA125, performance status, the presence and absence of ascites, histology, albumin, alkaline phosphatase, race and various molecular markers (e.g. her-2/neu) have been reported as important prognostic factors for women with the disease (Eisenhauer et al, 1999). Attempts have been made to construct a prognostic index of these factors (Van Houwelingen et al, 1989; Lund and Williamson, 1991; Ansell et al, 1993; Hogberg et al, 1993; Warwick et al, 1995; Brinkhuis et al, 1996), but these efforts have been hampered by the small size of available datasets. Large databases of ovarian cancer patients are required to establish reliably the effects of different prognostic factors on long-term outcome.

In this paper we use data from over 1100 women to develop a prognostic model for overall survival in ovarian cancer, assess its predictive performance, and discuss its application in clinical practice.

### PATIENTS AND METHODS

### Study population

Data from the Ovarian Cancer database of the ICRF Edinburgh Medical Oncology Unit, established in 1984, were used. Data were accrued from approximately 1400 ovarian cancer patients treated at the Western General Hospital, Edinburgh. The subset of 1189 patients with primary epithelial ovarian carcinoma was used. Patients were diagnosed between 01/01/1984 and 31/12/1999, and follow-up data were available up until the end of December 2000. For those patients lost to follow-up, vital status was checked in February 2001.

*Received 13 March 2001*
*Revised 23 May 2001*
*Accepted 5 July 2001*

*Correspondence to:* TG Clark

### Outcomes of interest

Overall survival, or equivalently time to death, was the outcome of interest. Information about recurrence was not felt to be robust so recurrence-free survival was not analysed.

### Prognostic factors

The possibly relevant prognostic factors considered in the initial stage of model-building process included age at diagnosis, FIGO stage, grade of tumour (I = well differentiated, II = moderately differentiated, III = poorly differentiated), histology, the presence or absence of ascites, the diameter of the largest residual tumour mass after primary cytoreductive surgery (< 2 cm, 2–5 cm and > 5 cm), performance status using the ZUBROD-ECOG-WHO scale (0 = normal activity, 1 = symptoms, but nearly ambulatory, 2 = some bed time, but needs to be in bed less than 50% of the normal daytime, 3 = needs to be in bed more than 50% of the normal daytime and 4 = unable to get out of bed), and the first CA125, alkaline phosphatase, and albumin laboratory test results that fell between diagnosis and 7 days after the first chemotherapy. As there was limited power to test for interactions, we proposed to investigate, globally, for interaction effects between the following prognostic factors: FIGO stage and log CA125 (Peters-Engl et al, 1999), FIGO stage and grade (Lund et al, 1990), FIGO stage and histology (particularly mucinous–Lund et al, 1990), and grade and histology (Carey et al, 1993).

### Statistical methods

Univariate analyses on continuous prognostic factors were performed using Cox regression and categorical prognostic factors were analysed using the Kaplan–Meier and the log-rank methods (Hosmer and Lemeshow, 1999). The linearity (or more complex forms) of the effect of continuous prognostic factors was assessed using fractional polynomials (Royston and Altman, 1994).

Because about half of the patients had at least one prognostic factor missing, multiple imputation (Rubin, 1987) was applied to account for missing prognostic factor data when considering all variables at once. Our approach, assuming that these data are *missing at random* (MAR) (Rubin, 1987), is similar to that described by Van Buuren et al (1999). This involved creating 10 complete datasets by replacing missing values with simulated values from a Gibbs sampling procedure (Gelfand and Smith, 1990). The analysis presented is a pooled summary of the results from the 10 datasets.

The fundamental method of multivariate analysis was Cox regression. The models were formulated by systematically removing predictors that were not significant ($P > 0.05$) starting from a (full) model containing all the prognostic factors (including factors that were non-significant in the univariate analysis). The proportional hazard assumption for each predictor was tested using an approximate score statistic of linear correlation between the rank order of failure times in the sample and Schoenfeld partial residuals (Grambsch and Therneau, 1994).

We evaluated the predictive performance of models by considering measures of discrimination and calibration. Discrimination refers to the ability to distinguish between high-risk and low-risk patients, and was quantified using the *c*-index and Nagelkerke $R^2$ ($R_N^2$) (Harrell, 1999). The *c*-index – a generalisation of the area under the Receiver Operating Characteristic (ROC) curve – is a probability of concordance between predicted and observed survival, with $c = 0.5$ for random predictions and $c = 1$ for a perfectly discriminating model. Similarly, a $R_N^2 = 0$ indicates no predictive ability and $R_N^2 = 1$ indicates perfect predictions. Calibration (or reliability) refers to whether the predicted probabilities agree with observed probabilities. Calibration was quantified using an estimate of slope shrinkage (Harrell, 1999), based on 200 bootstrap samples, and a value of 1 indicates perfect calibration.

All statistical analyses were carried out with S-Plus 2000 (Release 3) using the Hmisc, Design and MICE software libraries. The MICE library is available from www.multiple-imputation.com.

### RESULTS

### Patient characteristics

Patients were aged between 15 and 90 years at the time of diagnosis (median 61 years), presented at initial diagnosis with predominantly FIGO stages III and IV (64.6%), and predominantly with a serous papillary (51.8%) histology. The potential prognostic factors are summarised in Table 1. Performance status levels 3 and 4 were combined because of small numbers. CA125 and alkaline phosphatase laboratory results were skewed, and a summary of both transformed using natural logs is also presented. The median times between diagnosis and the CA125, alkaline phosphatase and albumin were 27 days (range 0–86 days), 30 days (range 0–88 days) and 30 days (range 0–88 days), respectively.

The treatment data are summarised in Table 2. 1140 (95.9%) patients underwent surgery, and of those, 860 (75.4%) had chemotherapy. Of the 893 (75.1%) patients who received chemotherapy as part of first-line treatment, 656 (73.5%) patients were treated with single-agent platinum regimens. The median time between diagnosis and first course of chemotherapy was 32 days (range 1–115 days). 1080 (94.7%) patients were diagnosed at time of surgery. Of those 49 (4.1%) patients who did not have surgery, 42 were FIGO stage III or IV, and 7 were missing a FIGO stage. Of those 296 (24.9%) patients who did not have chemotherapy, 96 (32.4%) were FIGO stage III or IV, 159 (53.7%) were FIGO stage I, and 10 were missing a FIGO stage.

### Long-term outcome

Follow-up information was available on all 1189 patients. 842 (70.8%) patients had died at the time of censoring the data. Median follow-up in the 347 (29.2%) patients who were living at the point of censoring was 1665 days (range 29–5852 days). 5-year survival in the cohort was 29.6% (95% Cl: 26.8–32.5%) in keeping with international mortality rates.

### Univariate analysis

Univariate analyses suggested that age, FIGO stage, the presence or absence of ascites, performance status, histology, debulking, albumin, grade, (log) CA125 and alkaline phosphatase were significant ($P \leq 0.05$) prognostic factors for overall survival. Investigations of linearity of albumin, age, alkaline phosphatase and CA125 in the univariate models suggested that alkaline phosphatase and CA125 required a natural log transformation. Missing data were not included in these analyses.

### Missing data

The frequency of missing values (labelled as 'unknown') for each prognostic factor is presented in Table 1. Overall, there were 2045 (17.2%) missing values distributed in 831 (69.9%) patients. 236 (19.8%) patients had 4 or more missing values, and only 4 (0.4%) patients with 7 or more missing prognostic factors. 1739 (85.0%) of the missing cells resulted from missing information on albumin, alkaline phosphatase, CA125 and performance status. The number of patients contributing to a complete case analysis using all the prognostic factors in Table 1 would be 358 (245 deaths), and contributing to the final model (Table 3) would be 450 (323 deaths). The missing data methodology produced values for the missing data that were consistent with the non-missing data, compensated for the uncertainty of producing that data, and ultimately has allowed us to perform an analysis with greater power on all 1189 patients.

### Multivariate analysis

Table 3 shows the results of the Cox regression analysis. There was significantly greater risk of mortality associated with older age, higher FIGO stage, poorer differentiated grade, the presence of ascites, worse performance status, greater residual disease, a lower albumin level and greater (log) alkaline phosphatase score. We combined FIGO stages III and IV because their (log) hazard ratios were identical to one decimal place ($P = 0.66$). Histology comparisons – which used the serous group as a reference – were

© 2001 Cancer Research Campaign

**Table 1** Potential prognostic factors

| **Prognostic variable** | ***N*** | **(%)** | ***n* = 1189 Median (range)** | ***P* value** |
|---|---|---|---|---|
| Age at diagnosis (years) | 1189 | (100.0) | 61 (15–90) | <0.001 |
| FIGO stage | | | | <0.001 |
| I | 281 | (23.6) | | |
| II | 119 | (10.0) | | |
| III | 590 | (49.6) | | |
| IV | 178 | (15.0) | | |
| Unknown | 21 | (1.8) | | |
| Grade | | | | <0.001 |
| I | 131 | (11.0) | | |
| II | 278 | (23.4) | | |
| III | 641 | (53.9) | | |
| Unknown | 139 | (11.7) | | |
| Histology | | | | <0.001 |
| Serous papillary | 616 | (51.8) | | |
| Endometrioid | 240 | (20.2) | | |
| Mucinous | 131 | (11.0) | | |
| Mesonephroid (clear cell) | 101 | (8.5) | | |
| Mixed mesodermal | 42 | (3.5) | | |
| Adenocarcinoma | 38 | (3.2) | | |
| Undifferentiated | 21 | (1.8) | | |
| Ascites | | | | <0.001 |
| Presence | 707 | (59.5) | | |
| Absence | 417 | (35.1) | | |
| Unknown | 65 | (5.5) | | |
| Performance status (Zubrog) | | | | <0.001 |
| 0 | 328 | (27.6) | | |
| 1 | 228 | (19.2) | | |
| 2 | 84 | (7.1) | | |
| 3+4 | 38 | (3.2) | | |
| Unknown | 511 | (43.0) | | |
| Debulking | | | | <0.001 |
| Complete Debulk (<2cms) | 641 | (53.9) | | |
| Partial Debulk (2–5 cms) | 165 | (13.9) | | |
| Non Debulk (>5cms) | 302 | (25.4) | | |
| Unknown | 81 | (6.8) | | |
| CA125 | 749 | (63.0) | 208 (6–22878) | – |
| Log CA125 | 749 | (63.0) | 5.3 (1.8–10.0) | <0.001 |
| Alkaline phosphatase (ALP) | 793 | (66.7) | 94 (26–1810) | – |
| Log ALP | 793 | (66.7) | 4.5 (3.3–7.5) | <0.001 |
| Albumin | 797 | (67.0) | 39 (20–50) | <0.001 |

statistically non-significant, except comparisons of the 'endometrioid', 'mixed mesodermal', and 'clear cell' groups with the 'serous' group ($P \leq 0.05$). There were also differences between the 'mixed mesodermal' and 'mucinous' and 'endometrioid' groups ($P < 0.05$). Using a likelihood-ratio test, an overall histology effect was significant ($P < 0.0001$), and therefore all histological comparisons were included in the final model. The only variable omitted was log CA125 which had a hazard ratio of 1.022 (95% Cl: 0.951–1.097) and was not statistically significant ($P = 0.54$). There were no significant overall interaction effects between FIGO stage and log CA125 ($P = 0.32$), FIGO stage and grade ($P = 0.74$), and grade and histology ($P = 0.06$). Although, there was no evidence of an overall FIGO stage and histology interaction ($P = 0.62$), there was weak evidence ($P = 0.03$) that those patients with a mucinous histology and advanced disease have more aggressive disease and worse prognosis than those with a serous histology (HR: 2.050; 95% CI: 1.073–3.916).

A slope-shrinkage (= 0.948) close to 1 indicates there is little need for recalibration and little evidence of over-fitting. Reasonably large values for the c-index (= 0.786) and Nagelkerke $R^2$ (=0.511) indicate that the set of prognostic factors is explaining the variation in outcome reasonably well, and this implies good prediction for individual patients. Figure 1 illustrates good discrimination ability for 4 risk groups constructed by partitioning using the quartiles of the patients' predicted risks based on the model. The survival curves and 95% confidence intervals for the risk groups are well separated. The *c*-index and Nagelkerke $R^2$ in those with early disease (FIGO stages I/II) was 0.715 and 0.250 respectively, whilst in advanced cases (FIGO stages III/IV), they were 0.727 and 0.371, respectively. This demonstrates that the model does discriminate well within groups with early or advanced disease.

### Application of the model

Our final prognostic model may be used to calculate expected survival probabilities at various times for different patients. Figure 2 is a nomogram (Lubsen et al, 1978) which enables a clinician to calculate a median survival time or 2-and 5-year survival probabilities for patients. For each level of the prognostic factors there is a

 *© 2001 Cancer Research Campaign*

**Table 2** Treatment

| | *n* = 1189 | |
|---|---|---|
| | ***N*** **or (median)** | **% or (range)** |
| Surgery | 1140 | 95.9 |
| Time from diagnosis to Surgery (days) | (0) | (0–56) |
| Chemotherapy | 893 | 75.1 |
| Single agents | | |
| Cisplatinum | 258 | 28.9 |
| Carboplatin | 246 | 27.5 |
| Chlorambucil | 135 | 15.1 |
| Other | 17 | 1.9 |
| Platinum-based combination | | |
| Cisplatinum / prednimustine | 83 | 9.3 |
| Platinum / taxane combinations | 38 | 4.3 |
| Cisplatinum / alpha interferon | 29 | 3.2 |
| Cisplatinum / topotecan | 28 | 3.1 |
| Carboplatin / hexalen | 27 | 3.0 |
| Cisplatinum / 5FU / hexamethylmelamine / prednisolone | 19 | 2.1 |
| Other | 11 | 1.2 |
| Non-platinum combinations | 2 | 0.2 |
| Time from diagnosis to chemotherapy (days) | (32) | (1–115) |
| Surgery and chemotherapy | 860 | 72.3 |

number of points allocated, and the total number of points from all prognostic factors can be converted into a median survival time or predicted survival probabilities at 2 and 5 years. For example, consider a 60-year-old woman (~36 points) with FIGO stage III (~39 points), a grade II tumour (= 13 points), a serous papillary histology (~8 points), no ascites present (= 0 points), a performance status of level 1 (~7 points), residual disease less than 2 centimetres (= 0 points), an albumin level of 30 (~14 points), and a log alkaline phosphatase level of log 90 (= 4.50) (~30 points). This patient has a total score of approximately 147, which translates into survival probabilities at 2 and 5 years respectively of approximately 0.7 and 0.25. The median survival time is approximately 2.5 years.

### DISCUSSION

Despite the plethora of published prognostic models in cancer, very few are used routinely in clinical practice (Wyatt and Altman, 1995). One problem associated with the acceptance of these models relates to their construction, which predominantly relies on small data sets. Sometimes limited analysable data are a result of low prevalence of disease and/or missing data. When performing a retrospective evaluation of large clinical databases, missing data can be problem. It is usual to exclude from an analysis those individuals on whom data are missing, and this practice leads to a reduction in the statistical power of an analysis and also leads to invalid results if the excluded group is a selective sub-sample of the entire dataset with respect to prognosis.

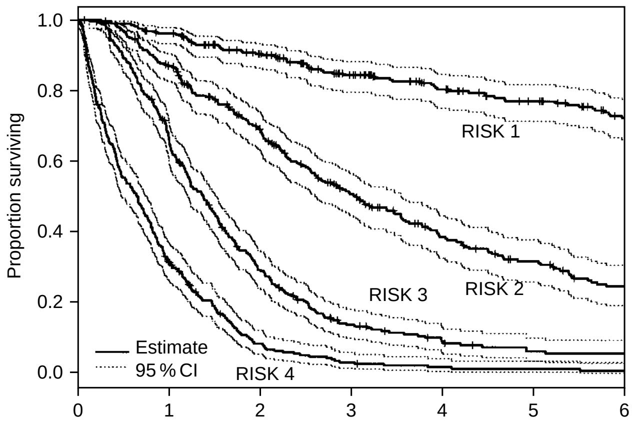

**Figure 1** Survival of risk groups. Based on the following cut-points of the linear predictions from the Cox model: –0.9922, 0.1105, and 0.9073

We applied a missing data methodology (multiple imputation) and Cox regression to construct a prognostic model for overall survival in 1189 ovarian cancer patients. This model included age, FIGO stage, ascites, performance status (2, 3 and 4 versus 0), grade, histology (particularly 'mixed mesodermal' versus other types), albumin, (log) alkaline phosphatase and debulking as (significant) prognostic factors ($P \leq 0.05$). Measures of predictive performance indicated that this model is well calibrated (i.e. has good ability to produce unbiased estimates of outcome), and has good discrimination abilities (i.e. has the ability to provide reasonably accurate predictions for individual patients). We also produced a nomogram for our model, which enables calculation of median survival times and expected survival probabilities for individual patients at 2 and 5 years.

Could the model be used in clinical practice? To help answer this question we have addressed 3 issues. First, we have reviewed previously published models – specifically the composition of models, sample size of studies used to construct the models, and ultimately their predictive ability. Second, we consider the generalisability and transportability of the model to other populations of ovarian cancer patients. Third, we comment on the practicability of applying the current model in a clinical setting.

### Existing models

We found many reports of multivariate models, but there are problems in synthesising this information into a meaningful overview. These problems include: small sample size of studies, limited details regarding which factors were considered in the analysis, inconsistencies in methodology between studies evaluating the same factor (e.g. categorisation of continuous factors or handling missing data), and non-uniform patient populations both between and within studies. The latter can be minimised when the data are from a prospective trial. Those analyses that are retrospective are often flawed, first, by considering response to treatment as a potential prognostic factor and, second, by not accounting for the use of different chemotherapy regimens.

We have restricted our review of prognostic models to those published since 1985 and with more than 200 patients in the study. This can be justified by potential problems with changes in treatment practice and small sample sizes. We have focused on variables that were available for analysis and their statistical significance in the model. There was no consideration of a variable's form or the magnitude of its hazard ratio(s). Table 4 is a summary of our results. The above limitations ignored, the most consistent 'statistically significant' prognostic factors are stage and post-operative residual disease. Histology and grade were incorporated into most assessments, but were less often significant. Some studies included either early or advanced ovarian cancer, while others included both groups. Although treatment practices may differ between early or advanced stage groups, it

*© 2001 Cancer Research Campaign* 

**Table 3** Multivariate prognostic model

| | ***n* = 1189, No. deaths = 842** | |
|---|---|---|
| **Prognostic factors** | **HR (95% CI)** | ***P* value** |
| Age (years) | 1.020 (1.012, 1.028) | <0.001 |
| FIGO stage | | |
| I | 1.000 | |
| II | 1.807 (1.299,2.514) | <0.001 |
| II+IV | 3.029 (2.299,3.991) | <0.001 |
| Grade | | |
| I | 1.000 | |
| II | 1.487 (1.070,2.067) | 0.017 |
| III | 1.560 (1.126,2.160) | 0.004 |
| Histology | | |
| Serous Papillary | 1.000 | |
| Endometrioid | 0.811 (0.657, 1.000) | 0.050 |
| Mucinous | 0.951 (0.697, 1.297) | 0.749 |
| Clear Cell | 1.381 (1.055, 1.807) | 0.019 |
| Mixed Mesodermal | 2.007 (1.392, 2.894) | <0.001 |
| Adenocarcinoma | 1.265 (0.833, 1.920) | 0.269 |
| Undifferentiated | 1.537 (0.941, 2.511) | 0.086 |
| Ascites | | |
| Absence | 1.000 | |
| Presence | 1.390 (1.171, 1.650) | <0.001 |
| Perf. Status | | |
| 0 | 1.000 | |
| 1 | 1.123 (0.897, 1.406) | 0.306 |
| 2 | 1.418 (1.072, 1.875) | 0.015 |
| 3+4 | 3.390 (2.231, 5.151) | <0.001 |
| Debulking | | |
| <2 cms | 1.000 | |
| 2–5 cms | 1.749 (1.398,2.189) | <0.001 |
| >5 cms | 1.836 (1.500, 2.248) | <0.001 |
| Log Alkaline Phosp. | 1.548 (1.301, 1.840) | <0.001 |
| Albumin | 0.979 (0.960, 0.999) | 0.036 |
| Model performance | Slope = 0.948 | *c*-index = |
| 0.786 | $R_N^2$ = 0.511 | |

HR = hazard ratio, $R_N^2$ = Nagelkerke $R^2$.

seems sensible to use all FIGO stages together in a dataset (if possible) because, given a larger magnitude of patients, multivariate analyses can detect heterogeneity in prognostic factor effects between the various stages by fitting interaction-terms.

In those studies (Swenerton et al, 1985; Hogberg et al, 1993; Hartmann et al, 1994; Kehoe et al, 1994; Kosary, 1994; Meden et al, 1995; Marx et al, 1997; Brun et al, 2000) that considered a complete (stage) case mix, grade and FIGO stage were consistently significant, with residual disease, histology, performance status, ascites, and albumin being significant in some, but not all models. The largest study (Kosary, 1994) used National Cancer Institute's Surveillance, Epidemiology, and End Results (SEER) data on 21 240 patients diagnosed between 1973 and 1987. Although, there was substantial power, data on residual disease and performance status were not presented. Among other large studies, Kehoe et al (1994) presented a study of 1184, but by removing patients with any missing data – an approach that could bias their results – the multivariate model was based on just 451 (38%) patients. Swenerton et al (1985) developed a model based on 556 patients with all FIGO stages, but their patients were treated with pre-platinum-based regimens.

Brun et al (2000) included a chemotherapy factor in their multivariate model as a way of adjusting for those patients with different chemotherapy regimens, and comparing those who received and did not receive chemotherapy. Although there was a statistically significant difference between those who received and did not receive chemotherapy, there was homogeneity between the effects of different chemotherapy regimens. In our multivariate model, the addition of a chemotherapy factor (applied versus not applied) was statistically significant ($P$ = 0.02). This is not unexpected as treatment decisions are based on baseline prognostic factors. For example, women with early stage disease are less likely to receive chemotherapy. This is confirmed in our cohort with the proportion of those treated with chemotherapy in FIGO stages I, II and III/IV being 43%, 74% and 88% respectively. We also considered the effects of different chemotherapy regimens in the modelling framework above, and found there was no difference in overall survival between single platinum and combination regimens ($P$ = 0.92). Although these are not randomised comparisons and interpretation is problematic, this result may be due to the high proportion (83%) of those chemotherapy patients receiving any form of platinum-based therapy, and the effect of less follow-up in the combination group (median 1.8 years). Fewer

*© 2001 Cancer Research Campaign*

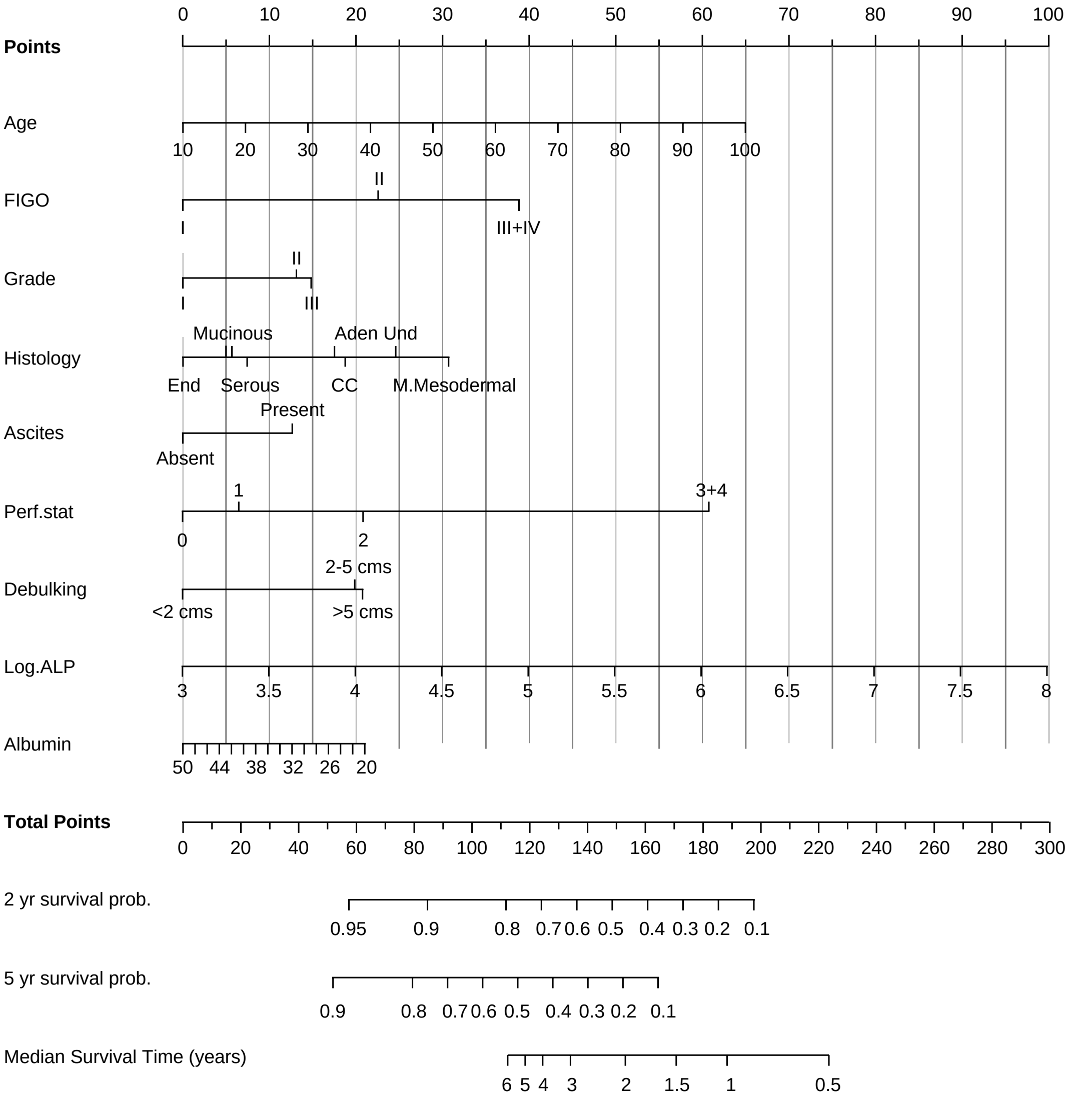

**Figure 2** Nomogram for median, 2 and 5 year survival. Aden = Adenocarcinoma, CC = Clear Cell, End = Endometrioid, Und = Undifferentiated, Perf.stat = Performance status, Log.ALP = log Alkaline Phosphatase than 5% of chemotherapy patients received new first-line regimens, such as taxanes, and in the future our model may have to account for the potential survival improvement from these and other therapeutic advances.

Given that 65% of our cohort were FIGO stage III or IV, it seems reasonable to compare our model with those from studies of advanced ovarian cancer. Our dataset ($n$ = 768) is one of the largest studies when considering these cases alone. A larger study, Marsoni et al, (1990), combined data from 4 clinical trials on FIGO stage III and IV patients. This study of 914 patients produced a model including age, FIGO stage, histology and residual disease. However, it was found that performance status could replace age and residual disease in those 721 patients not missing performance status. Of those other studies with more than 200 advanced cases (Marsoni et al, 1990; Alberts et al, 1993; Bruzzone et al 1995; Peters-Engl et al, 1999), the maximum size was 512 cases, and stage and residual disease were the core factors, with grade, histology, performance status, ascites, and albumin being in some, but not all models. We have not found a statistically significant difference between FIGO stages III and IV. Once again, there are studies with multivariate analyses performed using substantially fewer patients than those initially recruited to the study. Some studies (Van Houwelingen et al, 1989; Lund and Williamson, 1991; Kosary, 1994; Nagele et al, 1995; Peters-Engl et al, 1999; Brun et al, 2000) have dealt with missing data for each factor by including it as a separate level or category. This practice can lead to unrealistic hazard ratios and underestimation of standard errors (Greenland and Finkle 1995), but may be better than excluding whole cases.

CA125 was not significant in our model, and it is now widely accepted that baseline measurements do not predict long-term survival of patients, especially in patients with advanced ovarian cancer (Peters-Engl et al, 1999). There are some prognostic factors from Table 4 that are not present in our model. These include race, type of surgeon, and new molecular markers. Decreased survival has occasionally been observed in non-white women, and it is unknown whether this is due to racial differences in stage distribution, histology or grade or if there are possible questions concerning treatment differences, access to care or other socio-economic factors. Race has been an important prognostic factor in

*© 2001 Cancer Research Campaign* 

**Table 4** Selected multivariate prognostic models for overall survival in primary ovarian cancer with >200 patients in the study dataset and published since 1985

| Reference | No. patients | No. in final model | Diagnosis year (19XX) | Stage included | Rx | FIGO stage | Grade | Histology | Residuum | Age | Ascites | Performance status | CA125 | Albumin | Alkaline phosphat. | Other significant factors |
|---|---|---|---|---|---|---|---|---|---|---|---|---|---|---|---|---|
| 1 | 556 | <521 | 66–76 | All | C | * | * | – | * | – | – | * | | | | |
| 6 | 332 | 223 | 84–87 | All | P | * | * | – | * | * | | | | | | |
| 7 | 284 | 284 | 76–90 | All | M | * | * | – | | * | | | | | | |
| 8 | 1184 | 451 | 85–87 | All | M | * | * | – | * | * | – | | | | | Surgeon |
| 9 | 21240 | 21240 | 73–87 | All | ? | * | * | * | | * | * | | | | | Race, Node status |
| 12 | 266 | 266 | 82–92 | All | ? | * | * | – | – | * | | | | | | Her-2 |
| 15 | 251 | 251 | 82–92 | All | ? | * | * | – | – | * | | | | | | Her-2 |
| 17 | 287 | 287 | 75–95 | All | M | * | – | * | * | – | | | | | | Chemo |
| 13 | 201 | 201 | 84–93 | I | C,P | * | – | | | – | | | * | | | |
| 2 | 268 | 268 | 79–83 | II–IV | CP v CHAP | * | * | – | * | – | * | * | | | | Hospital |
| 4 | 301 | 301 | 81–86 | II–IV | CHAP | – | * | * | * | – | – | * | | | * | No. Metas |
| 10 | 512 | 512 | 82–94 | II–IV | P | – | * | – | * | – | | * | | | | |
| 14 | 362 | 213 | 81–91 | II–IV | P | – | – | – | * | – | | * | | * | | |
| 11 | 455 | <435 | 83–91 | III | M | * | * | * | * | * | * | | | | | |
| 3 | 914 | 852 | 78–86 | III–IV | P | * | – | * | * | * | | [*] | | | | |
| 5 | 342 | 291 | 85–89 | III–IV | C v P | * | | | | * | | * | | | | Race |
| 16 | 210 | 210 | 84–96 | III–IV | P | – | * | – | * | * | – | | – | | | |
| This Study | 1189 | 1189 | 84–99 | All | M | * | * | * | * | * | * | * | – | * | * | |

*Statistically significant (P ≤ 0.05), –not statistically significant, Rx chemotherapy regimen, ? data not presented, [*] if performance status included, age and residual disease omitted, P = Platinum based regimens, CP = cyclophosphamide and cisplatin, M = Mixed platinum and non-platinum regimens, C = cyclophosphamide, CHAP = cyclophosphamide, doxorubicin, hexamethylmelamine, cisplatin, 1 = Swenerton (1985), 2 = Van Houwelingen (1989), 3 = Marsoni (1990), 4 = Lund (1991), 5 = Alberts (1993), 6 = Hogberg (1993), 7 = Hartmann (1994), 8 = Kehoe (1994), 9 = Kosary (1994), 10 = Bruzzone (1995), 11 = Makar (1995), 12 = Meden (1995), 13 = Nagele (1995), 14 = Warwick (1995), 15 = Marx (1997), 16 = Peters-Engl (1999), 17 = Brun (2000).

© 2001 Cancer Research Campaign studies from the USA, but is less relevant in our homogenous 'Caucasian' Edinburgh cohort. Several studies have found that a trained gynaecologist and not a general surgeon should manage the surgery of a patient (Junor et al., 1999). This is not a concern in our dataset as more than 90% of the Edinburgh patients have been operated on by a gynaecologist. In the past few years there have been several reports about the prognostic impact of molecular markers, including oncogene products (her-2/neu, p21), tumour supressor gene products (p53, p16, pRB) and measures of drug sensitivity (Pgp, LRP, MRP, GST, BAX). There is difficulty in distilling these data into a clear conclusion on which of these measures are important prognostic factors, since there are few reports of substantial size. Two studies presented include her-2/neu, but we do not have sufficient data to test this factor. Although it is foreseeable that computer chip technology may produce new (molecular and genetic) prognostic factors and the prognostic models for the future are likely to be completely different, currently there is not enough data or follow-up to rigorously test them. The best way to do this is by large prospective studies, randomised if possible (Simon and Altman, 1994).

None of the studies in Table 4 formally assessed predictive ability using the calibration and discrimination measures we applied. Factors that affect predictive ability include: sample size, number of events, quality of the study design, quality of the data, and efficient model construction. Due to our sophisticated management of missing values, the model compares favourably to other studies with respect to sample size, number of deaths and power. Development of prognostic models is an exploratory analysis. By searching among many variables, there is a risk of including some variables that are not truly prognostic (Simon and Altman, 1994). During the construction of the model, we minimised false positive errors by assessing a limited number of predetermined variables. We also assessed assumptions associated with the multivariate Cox model, including the linearity of continuous variables and proportionality, and these were satisfied.

Some studies (Swenerton et al, 1985; Van Houwelinger et al, 1989; Lund et al, 1990; Marsoni et al, 1990; Lund and Williamson et al, 1991; Hogberg et al, 1993; Kappen and Neijt, 1993; Warwick et al, 1995) assessed discrimination by creating risk groups (via indices) based on the same methodology we employed. Log-rank methodology was applied to assess differences in these groups. As the construction of the risk groups is arbitrary, it is too difficult to compare them using measures of separation.

### Transportability to other populations

The Edinburgh population is predominantly Caucasian and, given the discussion of race above, our model may not be suitable outside this type of population. To assess the transportability of our model requires validation in at least one independent population (Altman and Royston, 2000). Table 4 shows that the variables in our model are broadly consistent with most of the other published models. In future work we plan to externally validate our model and a possible index. To our knowledge, 2 groups (Lund et al, 1990; Carey et al, 1993) have attempted to externally validate a model or index. Lund et al (1990) used Danish trial data to validate a statistical model from Dutch trial patients containing FIGO stage, grade, performance status, ascites, and residual disease. They found that performance status and residual disease were the only factors in common, and suggested that these factors would be a starting point for a basic index. Carey et al (1993) presented a larger validation of models from Dembo et al (1982), but their outcome of interest was ovarian cancer mortality. Overall survival is a more reliable outcome.

### Practicability of applying a model in a clinic

We have shown how a nomogram may be used to assess a woman's prognosis, and could potentially be used as the basis for making treatment decisions. However, any predictions would need to be presented with a measure of uncertainty, perhaps in the form of confidence limits. Our model, if and when validated, could benefit from being simplified and converted to a web-based medium for clinical utility.

In summary, we have constructed a prognostic model that is based on one of the largest analysable datasets in ovarian cancer. Our model is broadly consistent with other models in the literature, but it needs to be validated in an external dataset. In coming years the identification of prognostic molecular factors may change such models considerably. The production of valid models in the future will depend on the availability of large databases where both standard factors and molecular markers can be assessed. This could be done in the context of large (randomised) multicentre trials where treatment, follow-up and endpoints are predefined and measured according to identical criteria in all patients and, ideally, multiple laboratory measures are incorporated. We believe our model is one of the best available until such evidence is accumulated.

### ACKNOWLEDGEMENTS

We wish to thank Christina Davies, Victoria Cornelius and Sharon Love for reading earlier drafts of the manuscript.

### REFERENCES

Alberts DS, Dahlberg S, Green SJ et al (1993) Analysis of patient age as an independent prognostic factor for survival in a Phase III study of cisplatin-cyclophosphamide versus carboplatin cyclophosphamide in Stages III and IV ovarian cancer. *Cancer* **71**: 618–627

Altman DG, Royston P (2000) What do we mean by validating a prognostic model? *Stat Med* **19**: 453–473

Ansell SM, Rappoport BL, Falkson G et al (1993) Survival determinants in patients with advanced ovarian cancer. *Gynecol Oncol* **30**: 215–220

Brinkhuis M, Baak JPA, Meijer GA et al (1996) Value of quantitative pathological variables as prognostic factors in advanced ovarian carcinoma. *J Clin Pathol* **49**: 142–148

Brun J, Feyler A, Chene G et al (2000) Long-term results and prognostic factors in patients with epithelial cancer. *Gynecol Oncol* **78**: 21–27

Bruzzone M, Gadduci A, Brunetti I et al (1995) Prognostic factors affecting long-term survival of advanced ovarian cancer patients: univariate and multivariate analysis at 12 years of follow-up. *Proc Am Soc Clin Oncol* **14**: 812 (Abstr)

Carey MS, Dembo AJ, Simm JE et al (1993) Testing the validity of a prognostic classification in patients with surgically optimal ovarian cancer: a 15 year review. *Int J Gynecol Cancer* **3**: 24–35

CRC CancerStats (2000) *http://www.crc.org.uk/cancer/cancer_intro.html*, Cancer Research Campaign

Dembo AJ, Bush RS and Brown TC (1982) Clinico-pathological correlates in ovarian cancer. *Bull Cancer* **69**: 292–297

Eisenhauer EA, Gore M and Neijt JP (1999) Ovarian cancer: should we be managing patients with good and bad prognostic factors in the same manner? *Annals of Oncology* **10** (Suppl. 1): S9–S15

Gelfand AE and Smith AFM (1990) Sampling-based approaches to calculating marginal densities. *J Am Stat Assoc* **85**: 398–409

Grambsch P and Therneau T (1994) Proportional hazards tests and diagnostics based on weighted residuals. *Biometrika* **81**: 515–526

Greenland S and Finkle WD (1995) A critical look at methods of handling missing covariates in epidemiologic regression analysis. *Am J Epi* **142**: 1255–1264

© 2001 Cancer Research Campaign

Harrell FE (1996) Multivariate prognostic models: Issues in developing models, evaluating assumptions and adequacy, and measuring and reducing errors. *Stat Med* **15**: 361–387
Hartmann LC, Podratz KC, Kleeney GL et al (1994) Prognostic significance of p53 immunostaining in epithelial ovarian cancer. *J Clin Oncol* **12**: 64–69
Hogberg T, Cartensen J, Simonsen E et al (1993) Treatment results and prognostic factors in a population-based study of epithelial ovarian cancer. *Gynecol Oncol* **48**: 38–49
Hosmer DW and Lemeshow S. *Applied Survival Analysis: Regression modeling of time to event data*, Wiley, New York, 1999
Junor EJ, Hole DJ, McNulty L et al (1999) Specialist gynaecologists and survival outcome in ovarian cancer: a Scottish national study of 1866 patients. *Br J Obstet Gynaecol* **106**: 1130–1136
Kappen HJ and Neijt JP (1993) Neural network analysis to predict treatment outcome. *Ann Oncol* **4** (Suppl): 31–34
Kehoe S, Powell J, Wilson S et al (1994) The influence of operating surgeon's specialisation on patient survival in ovarian carcinoma. *Br J Cancer* **70**: 1015–1017
Kosary CL (1994) FIGO stage, histology, histologic grade, age and race as prognostic factors in determining survival for cancers of the female gynecological system: An analysis of 1973–87 SEER cases of cancers of the endometrium, cervix, ovary, vulva and vagina. *Sem Surg Oncol* **10**: 31–46
Lubsen J, Pool J and van der Does E (1978) A practical device for the application of a diagnostic or prognostic function. *Meth Inform Med* **17**: 127–129
Lund B and Williamson P (1991) Prognostic factors for overall survival in patients with advanced ovarian carcinoma. *Ann Oncol* **2**: 281–287
Lund B, Williamson P, van Houwelingen HC and Neijt JP (1990) Comparison of the predictive power of different prognostic indices for overall survival in patients with advanced ovarian carcinoma. *Cancer Research* **50**: 4626–4269
Makar AP, Baekelandt M, Trope CG et al (1995) The prognostic significance of residual disease, FIGO substage, tumor histology and grade in patients with FIGO stage III ovarian cancer. *Gynecol Oncol* **56**: 175–180
Marsoni S, Torri V, Valsecchi MG et al (1990) Prognostic factors in advanced epithelial ovarian cancer. *Br J Cancer* **62**: 444–450
Marx D, Meden H, Brune T et al (1997) Mib-1 evaluated proliferative activity in ovarian cancer with respect to prognostic significance. *Anticancer Research* **17**: 775–180
Meden H, Marx D, Raab T et al (1995) EGF-R and overexpression of the oncogene c-erbB-2 in ovarian cancer: immunohistochemical findings and prognostic value. *J Obstet Gynecol* **21**: 167–178
Nagele F, Petru E, Medl M et al (1995) Preoperative CA125: an independent prognostic factor in patients with stage I epithelial ovarian cancer. *Obstet Gynecol* **86**: 259–264
Peters-Engl C, Obermair A, Heinzl H et al (1999) CA125 regression after two completed cycles of chemotherapy: lack of prediction for long-term survival in patients with advanced ovarian cancer. *Br J Cancer* **81**: 662–666
Royston P, Altman DG et al (1994) Regression using fractional polynomials of continuous covariates: parsimonious parametric modelling. *Appl Statist* **43**: 429–467
Rubin DB (1987) Multiple Imputation for Nonresponse in Surveys. Wiley: New York
Simon R and Altman DG (1994) Statistical aspects of prognostic factor studies in oncology. *Br J Cancer* **69**: 979–985
Swenerton KD, Hislop TG, Spinelli J et al (1985) Ovarian carcinoma: A multivariate analysis of prognostic factors. *Obstet Gynecol* **65**: 264–269
Van Buuren S, Boshuizen HC and Knook DL (1999) Multiple imputation of missing blood pressure covariates in survival analysis. *Stat Med* **18**: 681–694
Van Houwelingen JC, Ten Bokkel Huinink WW, Van der Burg ATM et al (1989) Predictability of the survival of patients with ovarian cancer. *J Clin Oncol* **7**: 769–763
Warwick J, Kehoe S, Earl H et al (1995) Long-term follow-up of patients with advanced ovarian cancer treated in randomised trials. *Br J Cancer* **72**: 1513–7
Wyatt JC and Altman DG (1995) Commentary: Prognostic models: clinically useful or quickly forgotten? *Br Med J* **311**: 1539–1541

 *© 2001 Cancer Research Campaign*
